# Evaluating Hospital-Based Surveillance for Outbreak Detection in Bangladesh: Analysis of Healthcare Utilization Data

**DOI:** 10.1371/journal.pmed.1002218

**Published:** 2017-01-17

**Authors:** Birgit Nikolay, Henrik Salje, Katharine Sturm-Ramirez, Eduardo Azziz-Baumgartner, Nusrat Homaira, Makhdum Ahmed, A. Danielle Iuliano, Repon C. Paul, Mahmudur Rahman, M. Jahangir Hossain, Stephen P. Luby, Simon Cauchemez, Emily S. Gurley

**Affiliations:** 1 Mathematical Modelling of Infectious Diseases Unit, Institut Pasteur, Paris, France; 2 Centre National de la Recherche Scientifique, URA3012, Paris, France; 3 Center of Bioinformatics, Biostatistics and Integrative Biology, Institut Pasteur, Paris, France; 4 Department of Epidemiology, Johns Hopkins Bloomberg School of Public Health, Baltimore, Maryland, United States of America; 5 Influenza Division, Centers for Disease Control and Prevention, Atlanta, Georgia, United States of America; 6 Infectious Diseases Division, International Centre for Diarrhoeal Disease Research, Bangladesh, Dhaka, Bangladesh; 7 Discipline of Paediatrics, School of Women’s and Children’s Health, University of New South Wales, Sydney, New South Wales, Australia; 8 School of Public Health, University of Texas Health Science Center, Houston, Texas, United States of America; 9 Department of Lymphoma and Myeloma, The University of Texas MD Anderson Cancer Center, Houston, Texas, United States of America; 10 School of Public Health and Community Medicine, University of New South Wales Australia, Sydney, New South Wales, Australia; 11 Institute of Epidemiology, Disease Control and Research, Dhaka, Bangladesh; 12 Medical Research Council Unit The Gambia, Banjul, The Gambia; 13 Infectious Diseases Division, Stanford University, Stanford, California, United States of America; Harvard School of Public Health, UNITED STATES

## Abstract

**Background:**

The International Health Regulations outline core requirements to ensure the detection of public health threats of international concern. Assessing the capacity of surveillance systems to detect these threats is crucial for evaluating a country’s ability to meet these requirements.

**Methods and Findings:**

We propose a framework to evaluate the sensitivity and representativeness of hospital-based surveillance and apply it to severe neurological infectious diseases and fatal respiratory infectious diseases in Bangladesh. We identified cases in selected communities within surveillance hospital catchment areas using key informant and house-to-house surveys and ascertained where cases had sought care. We estimated the probability of surveillance detecting different sized outbreaks by distance from the surveillance hospital and compared characteristics of cases identified in the community and cases attending surveillance hospitals.

We estimated that surveillance detected 26% (95% CI 18%–33%) of severe neurological disease cases and 18% (95% CI 16%–21%) of fatal respiratory disease cases residing at 10 km distance from a surveillance hospital. Detection probabilities decreased markedly with distance. The probability of detecting small outbreaks (three cases) dropped below 50% at distances greater than 26 km for severe neurological disease and at distances greater than 7 km for fatal respiratory disease. Characteristics of cases attending surveillance hospitals were largely representative of all cases; however, neurological disease cases aged <5 y or from the lowest socioeconomic group and fatal respiratory disease cases aged ≥60 y were underrepresented.

Our estimates of outbreak detection rely on suspected cases that attend a surveillance hospital receiving laboratory confirmation of disease and being reported to the surveillance system. The extent to which this occurs will depend on disease characteristics (e.g., severity and symptom specificity) and surveillance resources.

**Conclusion:**

We present a new approach to evaluating the sensitivity and representativeness of hospital-based surveillance, making it possible to predict its ability to detect emerging threats.

## Abstract 2

Henrik Salje and colleagues propose a framework to examine the capacity of hospital-based surveillance data to detect public health threats in Bangladesh.

## Author Summary

**Why Was This Study Done?:**

- Many countries rely on hospital-based surveillance for the detection of infectious diseases of national and global public health relevance.
- It is often difficult to access suitable external reference data to assess the capacity of a surveillance system to detect cases and outbreaks or to characterize cases.

**What Did the Researchers Do and Find?:**

- We demonstrate a novel approach using healthcare utilization data to evaluate the sensitivity and representativeness of severe infectious disease surveillance in Bangladesh.
- The capacity to detect cases and outbreaks decreased with distance from surveillance hospitals.
- Cases captured by surveillance differed from cases in communities by age and socioeconomic status.
- Geographic coverage of surveillance could be improved by including other hospitals in the surveillance system.

**What Do These Findings Mean?:**

- The presented approach is applicable for a wide range of infectious diseases in different settings, taking some practical considerations into account.
- Hospital-based surveillance may have low sensitivity in rural areas at greater distances from surveillance hospitals, suggesting a risk of unrecognized transmission of emerging infectious diseases.
- Alternative surveillance strategies, such as including additional hospitals in the surveillance system or considering alternative data streams, may help to increase surveillance performance in such remote regions.

## Introduction

A well-functioning disease surveillance system is crucial for the identification and control of outbreaks, and hence the prevention of national and global health emergencies [1]. The World Health Organization (WHO) highlighted the value of national surveillance systems in the International Health Regulations (2005), an agreement among all member states to develop and maintain sufficient capacity for the detection, reporting, and control of public health threats of international concern [2]. Infectious disease surveillance should enable (i) the timely detection of outbreaks, (ii) the quantification of health problems, (iii) the identification of subpopulations at risk, and (iv) the assessment of temporal trends including the impact of control strategies [3,4].

National surveillance systems typically collect data from patients seeking care at sentinel hospitals or other healthcare facilities and can provide useful information for public health purposes. However, hospital-based surveillance generally underestimates disease burden since only a proportion of cases visit a hospital for care [5]. Low case detection may also undermine the value of hospital-based surveillance for outbreak detection. Moreover, if patients captured by the surveillance system are not representative of all cases in the community, surveillance statistics could lead to erroneous interpretations of disease patterns and misallocation of prevention resources. In particular, sex, socioeconomic status, or distance can affect healthcare seeking at hospitals, especially where access to care is limited [6–9]. Surveillance evaluation guidelines, such as those established by the US Centers of Disease Control and Prevention, list sensitivity and representativeness among the attributes that a public health surveillance system should possess and that require assessment [10,11]. In order to follow these guidelines, we need external reference data that are often unavailable in resource-poor settings [12].

Here, we present a new approach to evaluating the capacity of a surveillance system to detect and characterize disease cases, with emphasis on outbreaks of emerging infections that often occur as small case clusters in remote areas. We apply our methodology to assess hospital-based surveillance of severe neurological infectious disease and fatal respiratory infectious disease in Bangladesh.

## Methods

### Ethics Statement

The field teams obtained written informed consent from participants or their guardians (if <18 y of age) during community surveys. Healthcare utilization survey protocols were reviewed and approved by the Ethical Review Committee of the International Centre for Diarrhoeal Disease Research, Bangladesh.

### Protocol for Evaluating Sensitivity and Representativeness of Surveillance Systems

**Fig 1:**
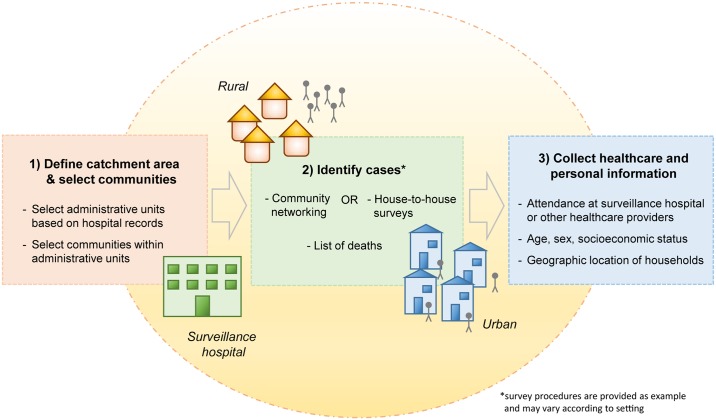
Key steps of the collection of healthcare utilization data to evaluate the sensitivity and representativeness of surveillance systems. In the Bangladesh example, the catchment areas of surveillance hospitals were first defined based on hospital records (e.g., areas where >50% or >75% of cases reside) [13,14]. Subsequently, small administrative units were chosen at random from within the catchment area, and all communities in the selected areas were surveyed. Cases in the community were identified based on lists of deaths in addition to community networking strategies (rural settings) or house-to-house surveys (urban settings). Information on symptoms (to establish case definitions), healthcare seeking behavior, and characteristics of cases was collected. In other settings, the exact survey procedures may vary according to the context.

Evaluating the sensitivity and representativeness of surveillance systems may be hampered by difficulties in identifying and characterizing the underlying case population. Here we describe how epidemiological studies can be used to identify cases with severe symptoms in communities and capture their personal and healthcare utilization characteristics (data collection stage) (Fig 1). In addition to detailing how we collected the data in this study, we provide information about how the approach could be varied in other settings. We subsequently demonstrate how such data can be used to evaluate the sensitivity and representativeness of surveillance systems (evaluation stage). We then apply our approach to the detection of severe neurological infectious diseases and fatal respiratory infectious diseases in Bangladesh as a case study.

### Data Collection

#### Selecting study locations

The first step was to randomly select communities at differing distances from the surveillance hospitals. We specified catchment areas of selected hospitals based on hospital records and subsequently randomly selected small administrative units from which all communities were surveyed. Selection of communities could also be done through census data or using detailed population maps of the area.

#### Identifying people with diseases in the selected community

Study teams visited the selected communities and identified cases that had had the disease of interest. The retrospective identification of severe disease cases in the community was based on syndromic criteria, used as a proxy for clinical case definitions that would be applied in healthcare facilities. The identification of such cases in the community is often the most problematic step, and the optimum strategy will depend on the local context, the severity of the disease, and the specificity of disease symptoms.

#### Collecting information on healthcare seeking and personal case characteristics

To estimate case detection probabilities, identify biases in case statistics, and characterize the healthcare utilization behavior in the population, we needed information about the healthcare seeking and personal characteristics of cases. In particular, we needed to identify whether the cases attended a surveillance hospital. Such information was obtained during household visits of identified cases. To understand the impact of distance from the hospital, we approximated the locations of households by the central positions of the small administrative units. Alternatively, household locations could be recorded precisely using GPS devices.

### Evaluation of the Surveillance System

#### Quantifying the probability of detecting a case

We estimated the case detection probability as the proportion of cases who reportedly attended a surveillance hospital among all cases identified in the community. We further assessed how this probability changed with distance from the surveillance hospital.

#### Quantifying the probability of detecting outbreaks

We subsequently used the estimated case detection probabilities to quantify the capacity of the surveillance system to identify disease outbreaks. We estimated outbreak detection probabilities for varying outbreak sizes and for outbreaks occurring at different distances from surveillance hospitals.

#### Assessing the representativeness of detected cases

We evaluated the representativeness of detected cases by estimating the difference between case statistics (proportions of specific case characteristics) based on all cases in the community and based on identified cases who attended the surveillance hospital. The investigated characteristics included sex, age, and socioeconomic status.

#### Assessing alternative surveillance strategies

To investigate how sensitivity and representativeness of the surveillance system could be improved by integrating other healthcare providers, we applied the evaluation procedures as described above to other healthcare provider types.

### Example Using Severe Neurological Infectious Diseases and Fatal Respiratory Infectious Diseases in Bangladesh

**Fig 2:**
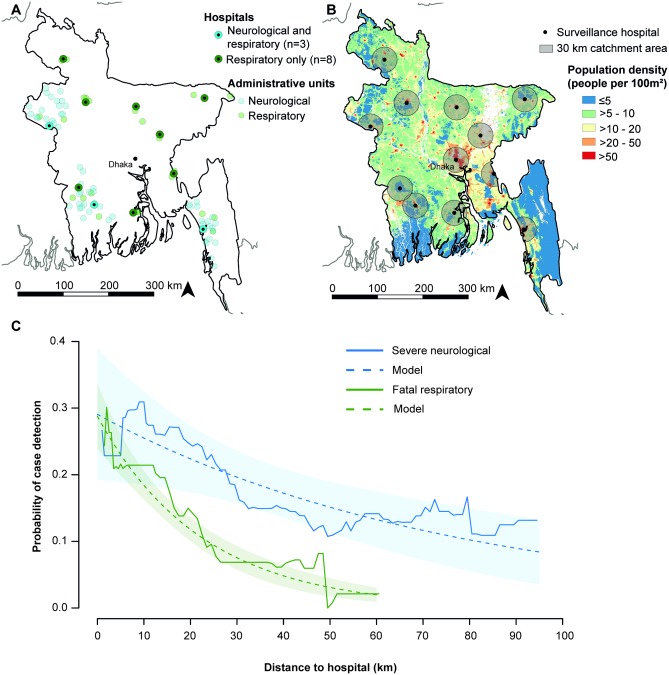
Location of administrative units and case detection probabilities by distance. (A) Location of surveillance hospitals and administrative units. The hospital in Dhaka City was excluded from the original studies. (B) Population density map of Bangladesh [16]. Sixty-eight percent of the population in Bangladesh lives >30 km from a surveillance hospital (including the Dhaka surveillance hospital), a distance at which case and outbreak detection probabilities are low. (C) Probability of surveillance case detection by distance. The observed probability was calculated as a moving average over a 25 km distance window. Case detection probabilities were estimated using log-binomial regression models including distance as an explanatory variable.

We demonstrate the application of the proposed evaluation strategy by using it to assess the capacity of hospital-based surveillance for severe infectious diseases in Bangladesh, which is based on tertiary care hospitals located throughout the country. We used data from two surveys carried out in catchment areas of some of these hospitals that investigated the healthcare utilization behavior of individuals with severe neurological infectious disease or fatal respiratory infectious disease (Fig 2A) [14,15]. These disease types are of great public health relevance in Bangladesh (e.g., Japanese encephalitis and influenza) but also represent symptoms typical of other emerging infectious diseases (e.g., Nipah and severe acute respiratory syndrome). A first survey collected data between 10 June 2008 and 30 March 2009 about cases with symptoms of severe neurological infection that occurred within the previous 12 mo in 60 small administrative units (mean population size of 28,000 people) in the catchment areas of three surveillance hospitals [14]. A second survey collected data between 3 April 2012 and 22 February 2013 about acute respiratory infection (ARI)–related deaths that occurred within the previous 24 mo in 22 administrative units in the catchment areas of 11 surveillance hospitals [15]. We considered ARI-related deaths as a proxy for respiratory disease of sufficient severity to require medical attention. The surveillance hospital in Dhaka City was excluded from the original studies because of the difficulty of defining the catchment area (a step necessary for the original study purpose), as people nationwide seek medical care in Dhaka. The surveys followed procedures as previously described and summarized below [13,14]. Characteristics of the study population are described in Fig. A in S1 Text.

### Community Healthcare Utilization Surveys

The catchment areas of selected hospitals were first specified based on hospital records (S1 Text). Small administrative units (mean population of 28,000 people) were subsequently selected randomly within the catchment areas, and all communities in the selected areas surveyed. The identification of cases in selected communities was based on social structures, i.e., cases were identified by visiting public meeting points, such as mosques, markets, or tea-stalls, where health problems in the community are often publicly discussed. Cases were subsequently confirmed by household visits. In urban areas, house-to-house surveys were conducted to compensate for less pronounced community structures. Additional fatal respiratory infectious disease cases were identified through lists of deaths provided by administrative officers. For both disease types, the identification of cases was based on syndromic criteria. We defined severe neurological infectious disease as fever with altered mental status for >6 h or with unconsciousness for ≥1 h, or fever with altered mental status, unconsciousness, or a new onset seizure that resulted in death. Fatal respiratory infectious disease (ARI-related death) was defined as having any two of the following symptoms in the 30 d prior to death: sudden onset of fever, cough, breathing difficulty, feeding difficulty, or runny nose. Deaths in children aged <5 y were also classified as ARI-related deaths if there was a sudden onset of breathing difficulty in the 30 d prior to death.

During surveys, information was collected on healthcare utilization behavior and personal characteristics of identified severe neurological and fatal respiratory disease cases. Cases or their household members were asked whether the case visited the surveillance hospital or any other healthcare provider, including other nonlocal hospitals, during his/her illness. Further, information on sex, age, socioeconomic status, and geographic location of households of cases was collected.

### Classification of Case Characteristics

We defined “community cases” as all severe neurological or fatal respiratory disease cases identified during community surveys (whether they attended a surveillance hospital or not) and “surveillance cases” as the subset of community cases who reportedly attended a surveillance hospital. For each case identified in community surveys, we identified whether they attended their nearest surveillance hospital. We then estimated the distance to that surveillance hospital as the distance between the residence administrative unit centroid and that specific surveillance hospital using QGIS [17]. Age was categorized as <5, 5–14, 15–59, and ≥60 y. A socioeconomic status index was generated by principal component analysis based on household assets (electricity, working television, bicycle, motorcycle, sewing machine, mobile phone) and categorized into tertiles (lowest, middle, and highest) [18]. In sensitivity analyses, we explored the use of continuous age and socioeconomic status classified into quintiles (S1 Text). Socioeconomic status was missing for 45 of 1,633 fatal respiratory disease cases, who were excluded from the analysis where this information was required. Three fatal respiratory disease cases were excluded from all analyses due to missing healthcare seeking information.

### Quantifying the Probability of Detecting Cases

We estimated the disease-specific case detection probability as the proportion of cases who reportedly sought care at a surveillance hospital among all cases identified during community surveys (number of surveillance cases/number of community cases) and computed 95% confidence intervals (95% CIs) based on the Clopper-Pearson exact method [19]. We quantified case detection probabilities by distance from a surveillance hospital using log-binomial regression analysis separately for severe neurological and fatal respiratory disease cases. We further investigated more complex functional forms of distance in log-binomial regression models. We fitted models with polynomial terms up to the fifth degree and models with basic splines with knots at various positions (between 20 and 50 km distance). Model fit was compared based on the Akaike information criterion (AIC), and the models with lowest AIC were selected. The fit of selected models was compared to the observed proportion of cases who attended surveillance hospitals at different distances (moving average over a distance window of 25 km). We estimated the proportion of the population living >30 km and >50 km from a surveillance hospital using gridded population density estimates of 100 × 100 m resolution [16].

### Quantifying the Probability of Detecting Outbreaks

To quantify the capacity of the surveillance system to detect outbreaks of varying sizes, we calculated the probability that at least one case was detected:

$${\text{Pr}}_{\text{outbreak}1}=1-{\left(1-\text{ Pr}\right)}^{s}$$

Pr_outbreak1_ is the outbreak detection probability based on a one-case threshold, Pr is the case detection probability, and *s* is the outbreak size. This calculation assumes that the probability of detecting a sentinel case is independent of other cases. We used distance-specific case detection probabilities estimated by log-binomial regression and obtained confidence intervals of outbreak detection probabilities based on the 95% CI limits of case detection probabilities. We further estimated the size of the smallest outbreak that would be detected with ≥90% probability by distance from the surveillance hospital. For emerging infectious diseases of global health importance, such as Nipah, severe acute respiratory syndrome, or avian influenza, a single detected case may be considered an outbreak. For other disease systems (e.g., endemic diseases or diseases for which differential diagnosis is difficult), an outbreak may be declared only after more than a single case is detected over a specified period of time and within specified geographic boundaries [20]. We can extend the framework to estimate the probability of identifying an outbreak with different outbreak thresholds applied, and we provide examples for outbreaks defined as detection of at least two cases or at least five cases. We calculated the probability of detecting at least two cases (Pr_outbreak2_) as one minus the probability of detecting no cases (Pr_0_) and exactly one case (Pr_1_):

$${\text{Pr}}_{\text{outbreak}2}=1-\left({\text{Pr}}_{0}+{\text{Pr}}_{1}\right)$$

Likewise, we estimated the probability of detecting at least five cases (Pr_outbreak5_) as one minus the probability of detecting no cases (Pr_0_) and exactly one (Pr_1_), two (Pr_2_), three (Pr_3_), and four cases (Pr_4_):

$${\text{Pr}}_{\text{outbreak}5}=1-\left({\text{Pr}}_{0}+{\text{Pr}}_{1}+{\text{Pr}}_{2}+{\text{Pr}}_{3}+{\text{Pr}}_{4}\right)$$

### Assessing the Representativeness of Surveillance Cases

We investigated the representativeness of surveillance cases (sex, age, and socioeconomic group) by comparing the proportion of cases with a specific characteristic (and exact binomial confidence intervals) among community cases to the proportion of cases with that characteristic among surveillance cases. We quantified the absolute difference in proportions (proportion of cases with characteristic among surveillance cases minus proportion among community cases) with 95% CIs and *p*-values using bootstrapping (2,000 bootstrap iterations) [21].

### Evaluating Alternative Surveillance Strategies

Based on the collected healthcare utilization data, we evaluated how the sensitivity and representativeness of a surveillance system may be improved by integrating other healthcare providers. We classified healthcare providers as (i) surveillance hospitals, (ii) other hospitals (government and private clinics), (iii) qualified private practitioners, and (iv) the informal sector (unqualified practitioners such as traditional healers, village doctors, homeopaths, and pharmacies). We estimated the proportion of cases attending each healthcare provider class, with exact binomial confidence intervals, and estimated outbreak detection probabilities based on proportions attending the surveillance hospital plus (i) other hospitals, (ii) qualified private practitioners, or (iii) informal healthcare providers. Furthermore, we compared the proportion of cases with each characteristic (sex, age, and socioeconomic group) among community cases to the proportion among those attending each healthcare provider class and quantified absolute differences in proportions with 95% CIs and *p*-values using bootstrapping (2,000 bootstrap iterations).

All statistical analyses and graphics were implemented in the R computing environment; maps were created using QGIS software [17,22].

## Results

The studied communities were located within 95 km (severe neurological infectious disease) and 62 km (fatal respiratory infectious disease) of a surveillance hospital. In these communities, 76 of 426 severe neurological disease cases (18%, 95% CI 14%–22%) and 234 of 1,630 fatal respiratory disease cases (14%, 95% CI 13%–16%) attended a surveillance hospital. Adjusting for distance, the case detection probability was nearly twice as high among severe neurological disease cases than among fatal respiratory disease cases (risk ratio 1.8, 95% CI 1.4–2.3; *p <* 0.001). At 10 km distance, an estimated 26% (95% CI 18%–33%) of severe neurological disease cases and 18% (95% CI 16%–21%) of fatal respiratory disease cases were detected by the hospital-based surveillance. The detection probability decreased with distance from the surveillance hospital, and the decline was faster for fatal respiratory disease than for severe neurological disease. A 10 km distance increase resulted in a 12% (95% CI 4%–19%; *p =* 0.003) relative reduction in case detection probability for severe neurological disease but a 36% (95% CI 29%–43%; *p <* 0.001) relative reduction for fatal respiratory disease (Fig 2C). Including more complex functional forms of distance in the log-binomial regression models did not improve model fit based on AIC (Table A and Figs. B and C in S1 Text).

**Fig 3:**
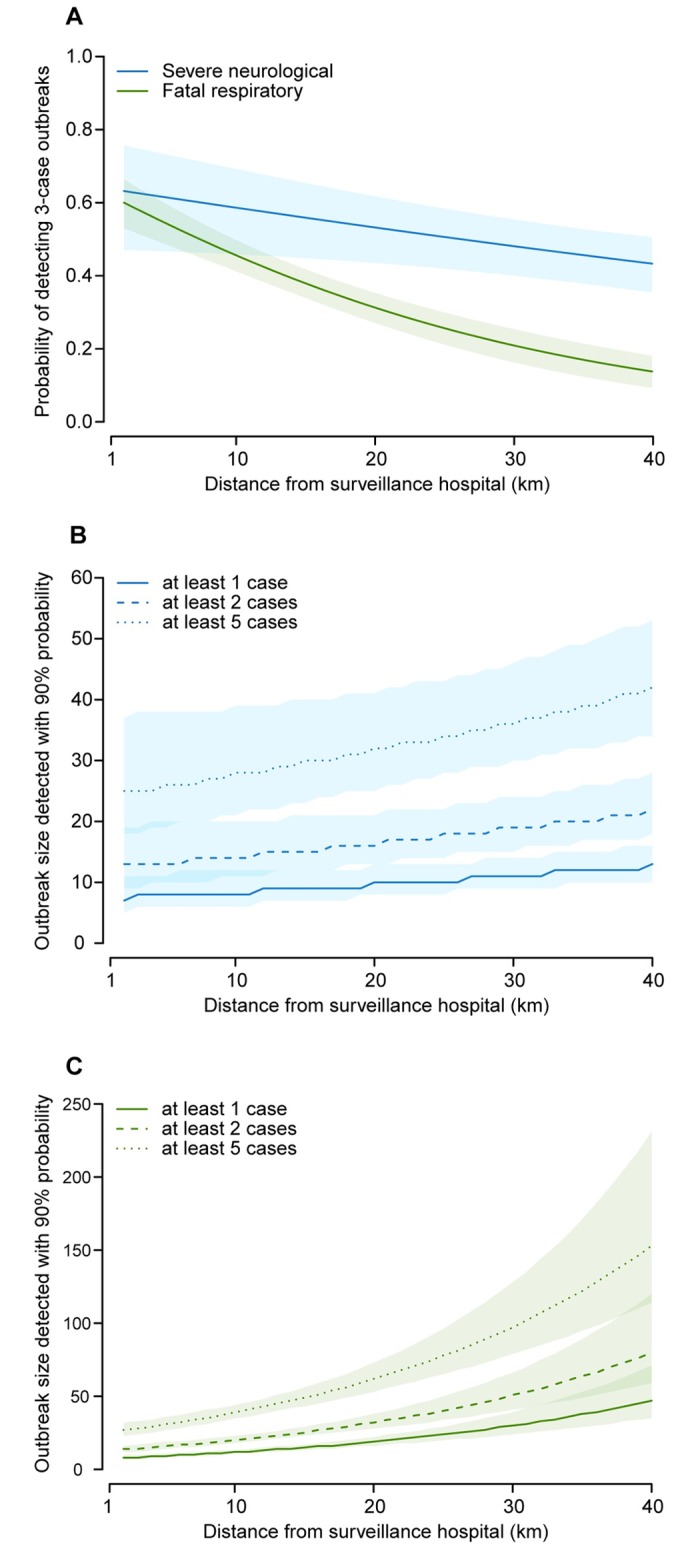
Outbreak detection capacity. (A) Probability of detecting outbreaks with exactly three cases of severe neurological or fatal respiratory disease by distance from surveillance hospital if a single detected case is considered an outbreak. (B) Smallest size of severe neurological disease outbreak that would be detected with ≥90% probability by distance from surveillance hospital for outbreak thresholds of at least one, two, or five detected cases. (C) Smallest size of fatal respiratory disease outbreak that would be detected with ≥90% probability by distance from surveillance hospital for outbreak thresholds of at least one, two, or five detected cases.

The probability of detecting an outbreak of exactly three cases (if a single detected case was considered an outbreak) dropped below 50% at distances greater than 26 km for severe neurological disease and at distances greater than 7 km for fatal respiratory disease (Fig 3A). Fig 3B and 3C show the minimum number of cases required for surveillance to detect outbreaks with a probability of ≥90% if different outbreak thresholds are applied. For outbreaks defined as detection of at least one case, we found that an outbreak of fatal respiratory disease required 12 cases (95% CI 11–13) to be detected with 90% probability at 10 km from a surveillance hospital, but 30 cases (95% CI 24–39) to be detected at 30 km. In contrast, the impact of distance on the outbreak size requirement was much more limited for severe neurological disease: eight cases (95% CI 6–12) at 10 km and 11 cases (95% CI 9–14) at 30 km. For outbreaks defined as detection of at least two cases, 14 severe neurological disease cases (95% CI 11–20) and 20 fatal respiratory disease cases (95% CI 18–23) would be necessary for an outbreak to be detected at 10 km distance, and 19 severe neurological disease cases (95% CI 15–24) and 51 fatal respiratory disease cases (95% CI 41–66) at 30 km. The necessary outbreak sizes increased further when a five-case threshold was applied, so that 28 severe neurological disease cases (95% CI 21–39) and 39 fatal respiratory disease cases (95% CI 35–44) would need to occur for an outbreak to be detected at 10 km distance, and 36 (95% CI 30–46) and 97 (95% CI 79–128), respectively, cases at 30 km.

**Fig 4:**
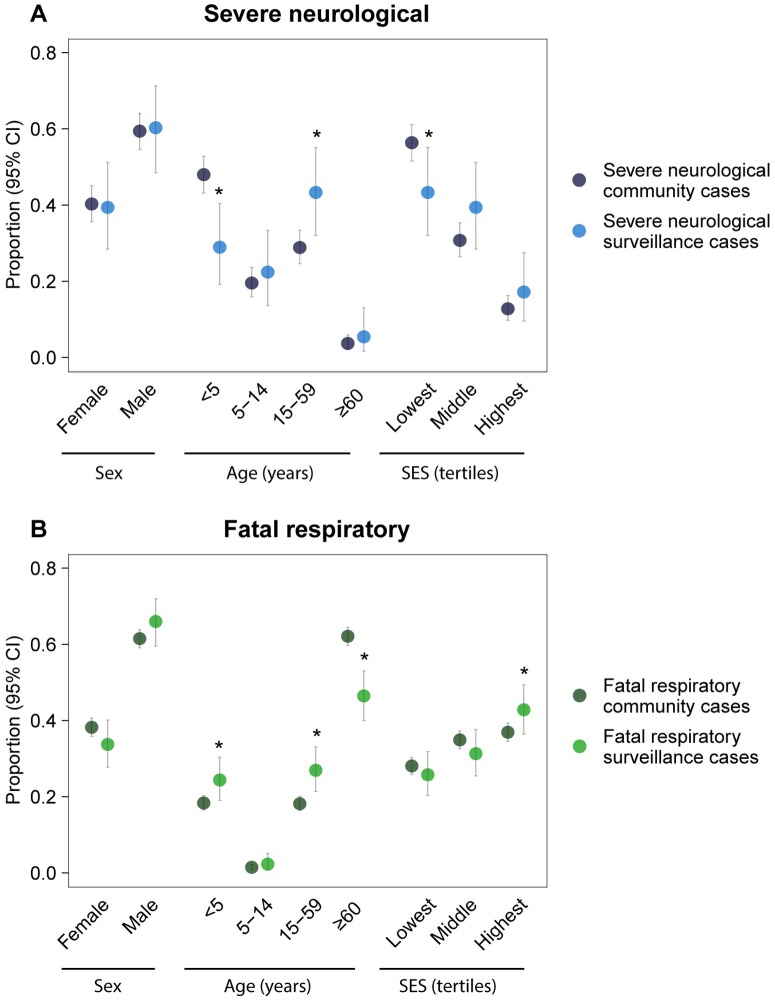
Representativeness of surveillance cases. Comparison of case statistics (proportion of cases with a characteristic) estimated for community cases to those estimated for surveillance cases for (A) severe neurological infectious disease and (B) fatal respiratory infectious disease. Significant differences (bootstrap *p* ≤ 0.05) are indicated with an asterisk. SES, socioeconomic status.

Surveillance hospital attendance among community cases varied by case characteristics, leading sometimes to biased disease statistics among surveillance cases (Table B in S1 Text). For severe neurological disease, individuals aged <5 y represented 48% of community cases but only 29% of surveillance cases (*p <* 0.001). Additionally, the proportion of cases in the lowest socioeconomic group was lower among surveillance cases than among community cases (43% versus 57%; *p =* 0.012), while the proportion of individuals aged 15–59 y was higher (43% versus 29%; *p =* 0.005) (Fig 4A). For fatal respiratory disease, the proportion of individuals aged ≥60 y (47% versus 62%; *p <* 0.001) was lower among surveillance cases than among community cases, while the proportion of individuals aged <5 y (24% versus 18%; *p =* 0.020), individuals aged 15–59 y (27% versus 18%; *p <* 0.001), and cases in the highest socioeconomic group (43% versus 37%; *p =* 0.022) was higher (Fig 4B). We observed a slight difference in the proportion of females for fatal respiratory disease (34% among surveillance cases versus 38% among community cases; *p =* 0.108), but not for severe neurological disease (39% versus 40%; *p =* 0.861). Results were consistent in sensitivity analyses with age as a continuous variable and socioeconomic status classified into quintiles (Figs. D and E in S1 Text).

**Fig 5:**
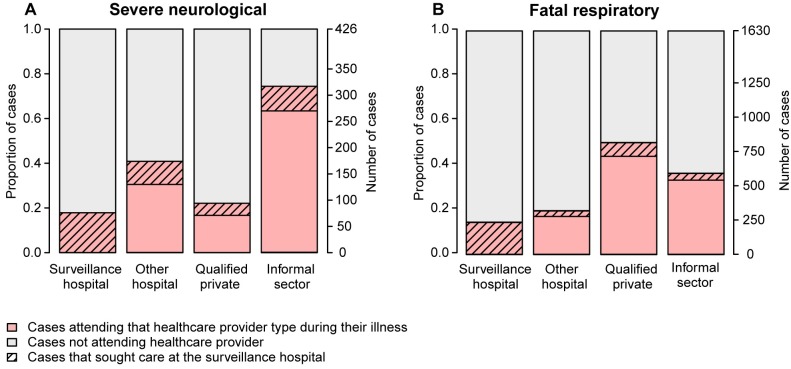
Attendance at surveillance hospitals and alternative healthcare providers. Proportion of (A) severe neurological and (B) fatal respiratory disease cases attending surveillance hospitals and other healthcare providers. Cases may attend several different healthcare providers during their sickness. Cases who attended a surveillance hospital at any time are indicated with diagonal hatching.

A substantial proportion of cases (severe neurological disease 42% [95% CI 38%–47%]; fatal respiratory disease 26% [95% CI 24%–28%]) visited multiple healthcare providers during their illness. Forty-eight percent (95% CI 44%–53%) of severe neurological disease cases and 31% (95% CI 29%–34%) of fatal respiratory disease cases attended any hospital, including surveillance hospitals (Fig 5). Including other hospitals that were attended by cases in the surveillance system could have increased the overall case detection probability by 31% (absolute increase) for severe neurological disease cases and 17% for fatal respiratory disease cases. The capacity to detect outbreaks would have increased, so that outbreaks containing four severe neurological or eight fatal respiratory disease cases would have been detected with ≥90% probability for any distance in the range 0–40 km from the original surveillance hospital, compared to 13 and 47 cases, respectively, with the current system (Fig. F in S1 Text). However, since individuals who attended any hospital had similar characteristics in terms of sex, age, and socioeconomic status as those attending surveillance hospitals (Fig. G in S1 Text), this expansion would not have increased disease detection in key groups such as the lowest socioeconomic group. Only with the informal sector incorporated in the surveillance system would cases in such groups be detected.

## Discussion

We described an analytic approach for evaluating the sensitivity and representativeness of hospital-based surveillance systems and applied it to surveillance for severe neurological diseases and fatal respiratory infectious diseases in Bangladesh. We quantified the proportion of cases detected and the probability that the surveillance system would detect different sized outbreaks by distance from the surveillance hospital. Finally, we characterized biases in surveillance statistics and identified potential improvements to the surveillance platform.

We estimated that approximately one-quarter of severe neurological disease cases and one-fifth of fatal respiratory disease cases occurring 10 km from a surveillance hospital would be detected with current surveillance. The proportion of cases attending a surveillance hospital declined significantly with increasing distance between individuals’ residence and the surveillance hospital, substantially faster for fatal respiratory disease than for severe neurological disease. These low detection probabilities mean that hospital-based surveillance in Bangladesh (like in most other resource-poor countries presumably) would likely miss a high proportion of single-case public health events. Of greater relevance is that surveillance system capacity to detect outbreaks and detection probabilities increased substantially with the number of cases. The required number of cases to detect outbreaks with high probability varied with disease type and distance from the surveillance hospital. It could be as low as about ten cases if the outbreak occurred <10 km from the surveillance hospital but increased quickly with distance for fatal respiratory disease. For outbreaks defined as a single detected case, we found that more than half of outbreaks with ten cases of fatal respiratory disease would be missed if the outbreak occurred >32 km from the hospital. Such detailed quantification of outbreak detection probability is essential to ascertain the likelihood that an emerging threat can be detected early enough to be contained [23].

In some circumstances, authorities may have to wait until more than a single case is detected to recognize that an outbreak is occurring. In particular, difficult differential diagnoses and lack of appropriate diagnostic tests mean that only when a number of cases are detected from the same area and over a short time frame will an outbreak be identified and further investigations conducted. In addition, where a low background level of transmission is expected (such as with endemic diseases), public health authorities may wait until a particular threshold is exceeded before declaring an outbreak. In both of these scenarios, where multiple cases need to be detected by the hospital before an outbreak is recognized, the optimal number of detected cases and their spatial and temporal separation will depend on the disease system. We can incorporate this information into our flexible framework and provide examples where we calculate the size an outbreak needs to be for scenarios where at least two or five cases need to be detected (Fig 3B and 3C). In particular, this demonstrates that if an outbreak is identified only once five cases are detected at the surveillance hospital, the size of the outbreak would have to be substantially larger (e.g., nearly 100 total cases of a fatal respiratory disease at 30 km from a surveillance hospital) for there to be a 90% chance of an outbreak being identified. This highlights the possibility that, by the time an outbreak reaches sufficient size to be detected by the system, outbreak control measures may be much less effective at controlling spread. Thresholds for case counts that trigger an outbreak response should be crafted taking this possibility into account.

Low detection probabilities for outbreaks that occur far from surveillance hospitals are an important concern because pathogens with high case fatality such as Japanese encephalitis and Nipah virus are nearly exclusively found in rural communities in Bangladesh [24,25], and these communities are usually located far from surveillance hospitals. Rural environments are also considered to be at highest risk for the emergence of novel pathogens [26,27]. Population distribution maps suggest that 68% of the population in Bangladesh live in communities >30 km from a surveillance hospital (representing 108 million individuals) and 40% live >50 km from a surveillance hospital (representing 63 million people) (Fig 2B). Strengthening healthcare-based surveillance in these areas should be a priority, and cost-effective approaches to achieving surveillance targets need to be identified. There is increasing recognition of the value of novel data sources to improve the sensitivity of infectious disease surveillance, some of which can provide crucial information in remote areas [20]. Novel approaches include surveillance for media reports of disease clusters, as used for various infectious diseases in Bangladesh [12,28], and training of local drug sellers to recognize and report disease symptoms, as rolled out nationally to enhance tuberculosis surveillance in Ghana [29]. Other surveillance data streams, such as monitoring over-the-counter medication sales, telephone triage, and web-based queries, have been successfully integrated in surveillance systems in resource-rich settings [30].

We found that cases attending surveillance hospitals were not necessarily representative of all cases in the community. In particular, the youngest severe neurological disease cases and the oldest fatal respiratory disease cases were less likely to attend surveillance hospitals, and attendance was also lower among cases in the lowest socioeconomic group. Similar variation in hospital attendance has been reported in other resource-poor settings [6,8,9], indicating that hospital-based surveillance in these countries may have comparable limitations. Disease statistics obtained through hospital-based surveillance have to be interpreted in the light of detected biases, and correction factors may need to be applied. For example, underestimating severe neurological disease among young children may mislead any future Japanese encephalitis vaccination strategy [31,32]. Differential surveillance hospital attendance may also influence the capacity to detect emerging infections, such as the avian influenza A (H7N9) virus that emerged in 2013 in China with observed cases mainly among elderly men [33].

Overall, access to appropriate care was poor—over 30% of community cases with severe disease or who died in our study never saw a qualified provider. Such low access is a common problem in low-income settings and means that a large proportion of the population, and particularly subgroups that are potentially at highest need, do not receive the required medical attention [6–9]. For example, difficulties accessing qualified healthcare providers for elderly people, who are often at greatest risk of respiratory disease, can have severe consequences for the outcome of disease. Previous studies have demonstrated that accessibility to healthcare is a significant predictor of morbidity and mortality among elderly individuals with respiratory disease [34].

The study showed that healthcare utilization behavior varied by disease type, which may be due to different characteristics of cases such as their age, socioeconomic status, and geographic location (Fig. A in S1 Text). The majority of fatal respiratory disease cases were ≥60 y old and may have faced limitations in mobility; moreover, rapid progression of disease to death may have prevented cases in this age group from seeking appropriate care. Cases and their family members in general may have also perceived neurological symptoms as more severe, resulting in higher motivation to attend a qualified healthcare provider [7].

We evaluated potential improvements of surveillance by analyzing healthcare seeking behavior among cases identified in communities. While the majority of individuals did seek care, much of this was in the informal sector, which cannot easily be incorporated into surveillance activity. Nevertheless, including other hospitals attended by cases in the surveillance system (the exact location and number of these hospitals was unfortunately not identified during surveys) would double case detection probabilities and allow detection of medium-sized outbreaks (<10 cases) in a wider geographic area. However, in the case of Bangladesh, such extension is likely to be prohibitively expensive. Mapping other hospitals in Bangladesh that may serve as surveillance sites would allow testing of various surveillance scenarios to identify the optimal location of surveillance sites while keeping the same total number or to quantify the number of sites needed to achieve a target surveillance coverage [35]. Many emerging infectious diseases originate as spillover infections of zoonotic diseases into the human population [36]. Therefore, mapping the occurrence of relevant zoonotic diseases (e.g., avian influenza) and combining such maps with the estimated outbreak detection probabilities would allow highlighting of surveillance gaps for particular types of emerging infectious diseases.

The capacity of surveillance systems to detect outbreaks will depend not only on the probability that a case attends a surveillance hospital, but also on whether the case undergoes confirmatory laboratory testing and is subsequently reported through the surveillance system by the hospital. Here we assumed a “best-case scenario” with a fully functional surveillance system at the hospital level, where each case who attends the surveillance hospital is ultimately recognized and confirmed as a case. Case detection at the hospital may however be incomplete, since case definitions at hospitals may differ from syndromic definitions, a surveillance sampling frame may be applied, or resources and trained personnel for the diagnosis and reporting of cases may be limited [37]. The calculation of case and outbreak detection probabilities may be adjusted for misdiagnosis and underreporting at hospitals if such information is available. We further assumed complete detection of cases in communities during surveys. Although a few cases may have been missed, this assumption is justified as we investigated severe disease conditions that are easily remembered by family and community members. Moreover, survey procedures combining interviews of key informants and house-to-house visits were specifically designed to capture near-complete case information. Further, any missed cases are unlikely to impact our estimates, as such impacts would occur only if there was differential healthcare seeking between those detected and those missed. We investigated spatial differences in hospital attendance based on the straight-line distance of communities from the surveillance hospital. If available, other distance measures such as travel distance or travel time may provide a more accurate indicator of distance from the surveillance hospital. In some cases, these measures may strongly vary with the season, and it would be interesting to explore how that may impact the probability of detecting an outbreak. We assumed that cases did not visit other surveillance hospitals than the catchment hospital. Given the poor road infrastructure in the country, it would be very unusual to travel to a tertiary care hospital that was not the closest one. It is possible that some individuals traveled to Dhaka; however, these are likely to be wealthier individuals who would visit small private healthcare facilities that are not part of the surveillance network. The surveillance hospital in Dhaka was not included in our study. This is unlikely to have biased our assessment of the performance of the surveillance system outside the capital. Indeed, this would introduce a bias only under the unlikely scenario that many cases in our study who did not attend the nearest surveillance hospital (and were therefore not captured there) instead attended the surveillance hospital in Dhaka (and were captured there). Surveillance system performance in the capital city may however differ from elsewhere, and a comprehensive assessment of the national surveillance system would therefore have to include Dhaka. Moreover, hospital-based surveillance is only one surveillance type in Bangladesh, and other data sources need to be considered to assess the country’s overall capacity to detect public health events.

The described methodology is applicable to assessing surveillance for other severe diseases in resource-poor settings, keeping in mind practical constraints. Conducting community surveys may be labor intensive, time consuming, and expensive depending on the setting and may be particularly challenging in densely populated areas such as Dhaka. Nonetheless, such surveys are valuable tools for obtaining external reference data and simultaneously assess heterogeneities in healthcare access. The effectiveness of community networking may depend on the social structures in the study area; where social links are weaker (e.g., in urban areas), house-to-house surveys, even though more labor intensive, may be more suitable for the identification of cases in the community. The proposed strategy is valid for diseases of sufficient severity to require medical attention and to be remembered by cases and family members. The approach is syndromic (i.e., disease types are classified based on a set of symptoms), and the classification specificity may vary by disease.

In conclusion, this study allowed us to quantify the sensitivity and representativeness of hospital-based surveillance and to identify weaknesses, particularly in detecting small- to medium-sized outbreaks in remote areas. These findings highlight difficulties that low-middle-income countries may have in meeting International Health Regulations requirements, despite considerable investment in hospital-based surveillance platforms.

## Supporting Information

S1 Dataset Healthcare utilization by distance from surveillance hospital. (XLSX)

S2 Dataset Healthcare utilization by case characteristics. (XLSX)

S1 Text Additional results and figures. (PDF)

## Abbreviations

AIC: Akaike information criterion
ARI: acute respiratory infection
WHO: World Health Organization
