# Supplementary material for: Evaluating Hospital-Based Surveillance for Outbreak Detection in Bangladesh: Analysis of Healthcare Utilization Data
Source: PLoS Med. 2017 Jan 17;14(1):e1002218. doi: 10.1371/journal.pmed.1002218 (PMC5240927; doi:10.1371/journal.pmed.1002218)
Supplement: S1 Text — (PDF) [file pmed.1002218.s003.pdf]

S1 Text

Selection of communities in Bangladesh

The catchment area of selected hospitals was first specified based on hospital records as districts where more than 50% (severe neurological) or 75% (fatal respiratory) of admitted cases reside. Small administrative areas (mean population of 28,000 people) were subsequently selected randomly within the catchment areas and all communities in the selected areas surveyed.

Characteristics of the study population

Community surveys identified 426 cases of severe neurological disease (969 cases of neurological disease of any severity) and 1,633 cases of fatal respiratory disease. Three fatal respiratory cases were excluded from the analysis due to missing healthcare utilization data. Households of severe neurological disease cases were located within 1-95 km distance from their catchment hospital (median 39 km; interquartile range (IQR) 21-60) and households of fatal respiratory disease deaths within 3-62 km (median 13 km; IQR 7-32) (Main text Fig 1 A). For both disease types, the proportion of male cases identified in the community was higher than females (60% for severe neurological disease and 62% for fatal respiratory disease) (Fig A). Severe neurological cases were generally younger than fatal respiratory cases (median age of 5 vs. 65 years). Furthermore, severe neurological cases belonged to a lower socioeconomic class; the majority of severe neurological cases (57%) were of the lowest socioeconomic group compared to fatal respiratory disease cases of whom only 28% were of lowest status.

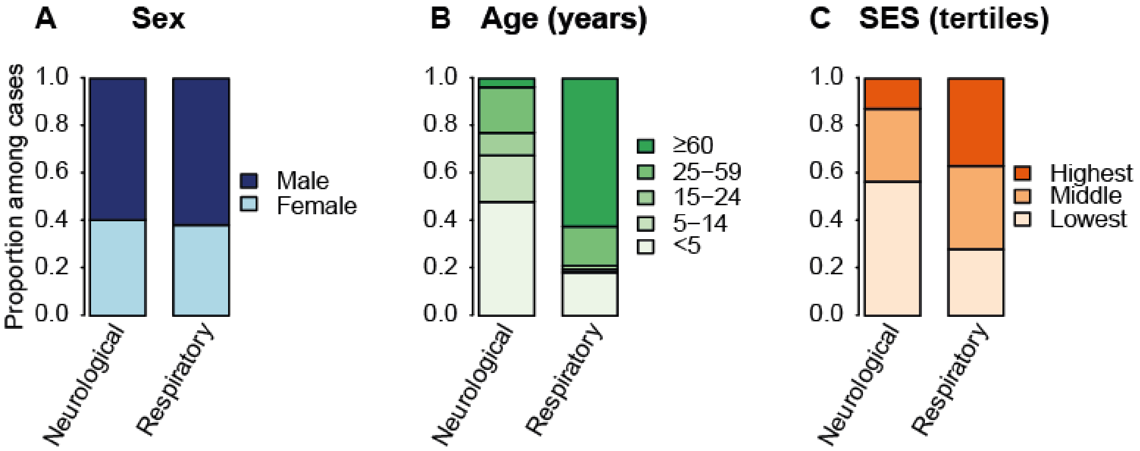

Fig A. Characteristics of the study population. Sex **(A)**, age **(B)**, and socioeconomic status (SES) **(C)** of severe neurological and fatal respiratory cases identified in the community.

Estimating case and outbreak detection probability by distance - alternative models

We investigated more complex functional forms of distance in log-binomial regression models, such as polynomial terms up to the 5th degree or basic splines with knots at various positions (between 20 and 50 km distance). Model fit was compared based on the Akaike information criterion (AIC) and models with lowest AIC were selected. The results of the model selection procedure are summarized in Table A. While including a squared distance term led to better optical fit for severe neurological case detection probabilities (Fig B), no improvement of model fit was observed based on the AIC (AIC of 394 for model 1- a log-binomial regression model including only distance as explanatory variable vs. 396 for model 2- the model including additionally a squared distance term). We further explored if outbreak detection probabilities were influenced by model choice and changes in detection probabilities were only minimal (Fig C). Based on model 2 we estimated that the probability of detecting outbreaks of three severe neurological cases was 60% at 10 km distance (compared to 59% with model 1) and 46% at 30 km distance from surveillance hospitals (compared to 47% with model 1). Outbreak sizes detected with ≥90% probability were of 8 at 10 km distance (8 with model 1) and 12 at 30 km distance (11 with model 1). We further explored the use of basic splines with knots at various locations, however did not detect model improvement based on AIC (Table A).

Model	AIC severe neurological	AIC fatal respiratory
Model 1: distance	394	1264
Model 2: polynomial distance term, degree 2	396	1266
Model 3: polynomial distance term, degree 3	398	1268
Model 4: polynomial distance term, degree 4	396	1270
Model 5: polynomial distance term, degree 5	398	1267
Model 6: distance, knot 20km	396	1266
Model 7: distance, knot 30km	396	1266
Model 8: distance, knot 40km	395	1268
Model 9: distance, knot 50km	396	1266
Model 10: distance, knots 20km+40km	396	1268
Model 11: distance, knot 30km+40km	396	1268

Table A. Comparison of models to predict case detection probabilities by distance. Model selection was based on the lowest AIC, which for both disease types was the log-binomial regression model including distance as linear term (model 1).

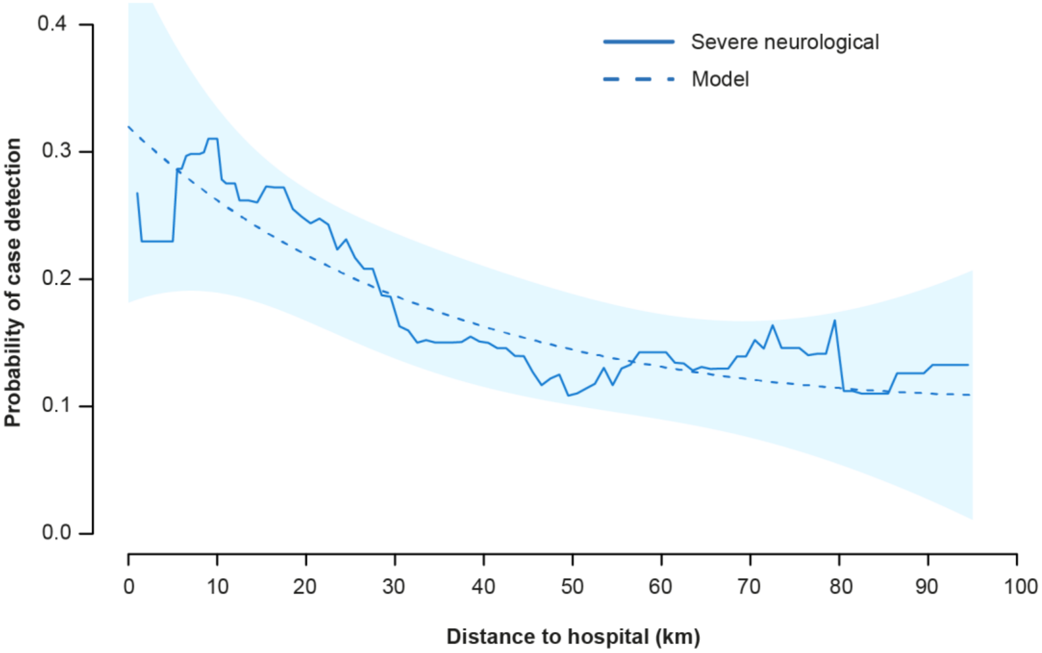

Fig B. Case detection probability for severe neurological disease estimated based on a log-binomial regression model including distance and squared distance as explanatory variables.

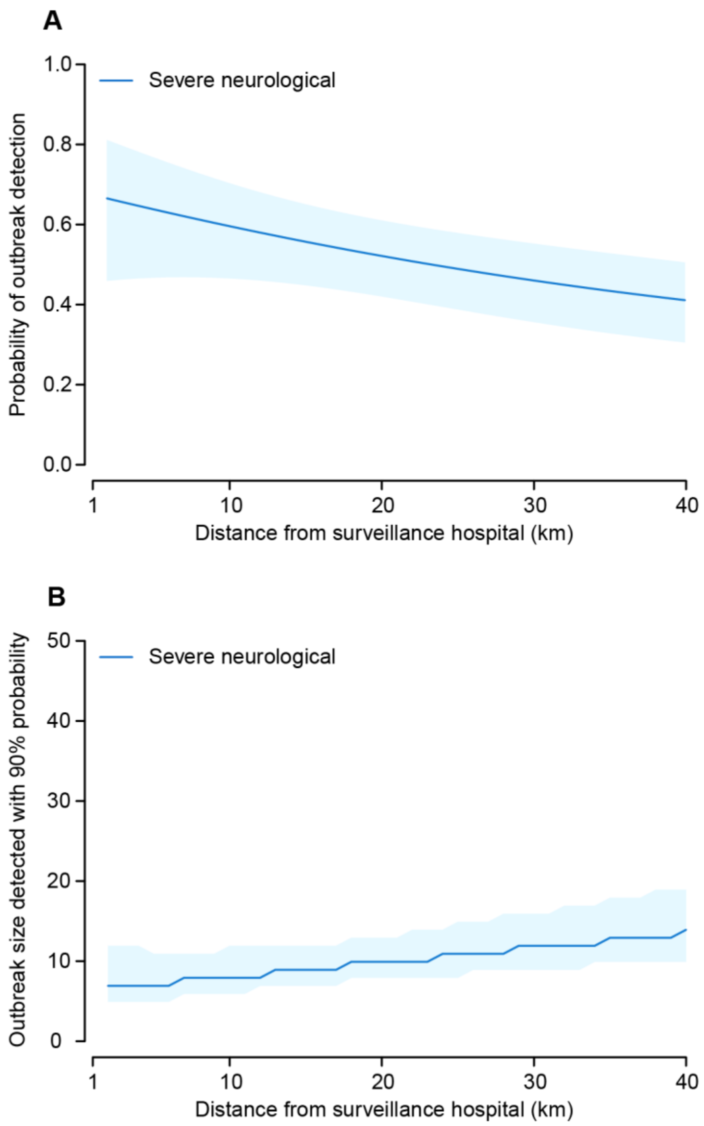

Fig C. Probability of detecting an outbreak of three cases of severe neurological disease (outbreak threshold of at least one detected case) **(A)** and outbreak size necessary for 90% detection probability (**B**) based on case detection probabilities estimated by log-binomial regression models including distance and squared distance as explanatory variables.

Case detection bias by age

We investigated reporting probabilities and detection bias by age. Fig D shows detection probabilities of severe neurological and fatal respiratory infections by age estimated by basic spline regression (age basic splines of degree 4 for severe neurological and degree 2 for fatal respiratory disease in logistic regression models) and the proportion of all community and surveillance cases within a moving 5-year age window (at the midpoint of the window). While the youngest severe neurological cases were underrepresented among surveillance cases, the oldest fatal respiratory cases were underrepresented among surveillance cases. For presentational purpose, results are summarized for age categories (<5, 5-14, 15-59, and ≥60 years) in the main text.

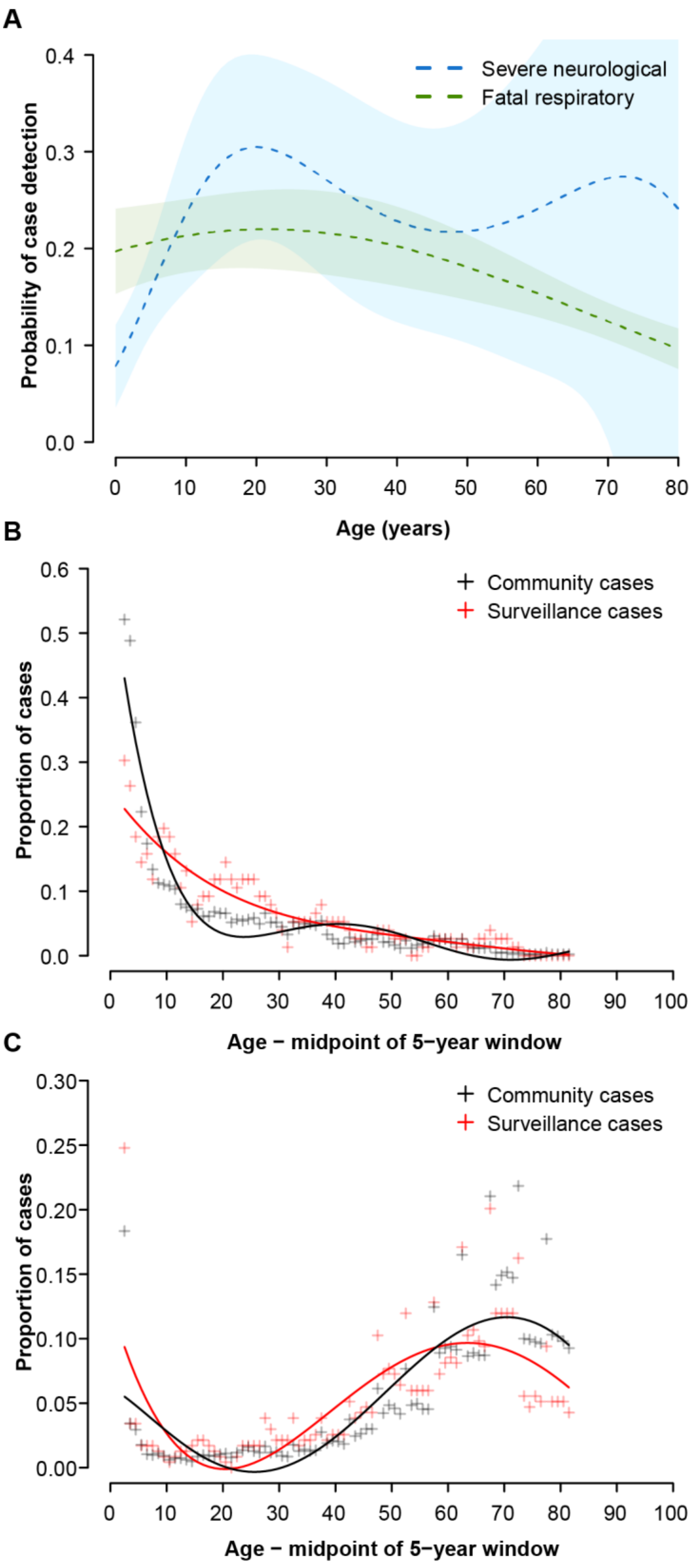

Fig D. Case detection probability by age estimated by spline regression **(A)**. Proportion of severe neurological **(B)** and fatal respiratory **(C)** cases within moving 5-year age groups. The proportion was estimated as proportion of all community cases or surveillance cases that fall within the moving 5-year window.

Case detection bias by socioeconomic status quintiles

To investigate if classification of cases into socioeconomic status tertiles has influenced the results presented in the main text, we further investigated case detection by quintiles (Fig E). Results are consistent with the tertile analysis, where severe neurological cases of low socioeconomic status were underrepresented among surveillance cases while fatal respiratory cases of high socioeconomic status were overrepresented.

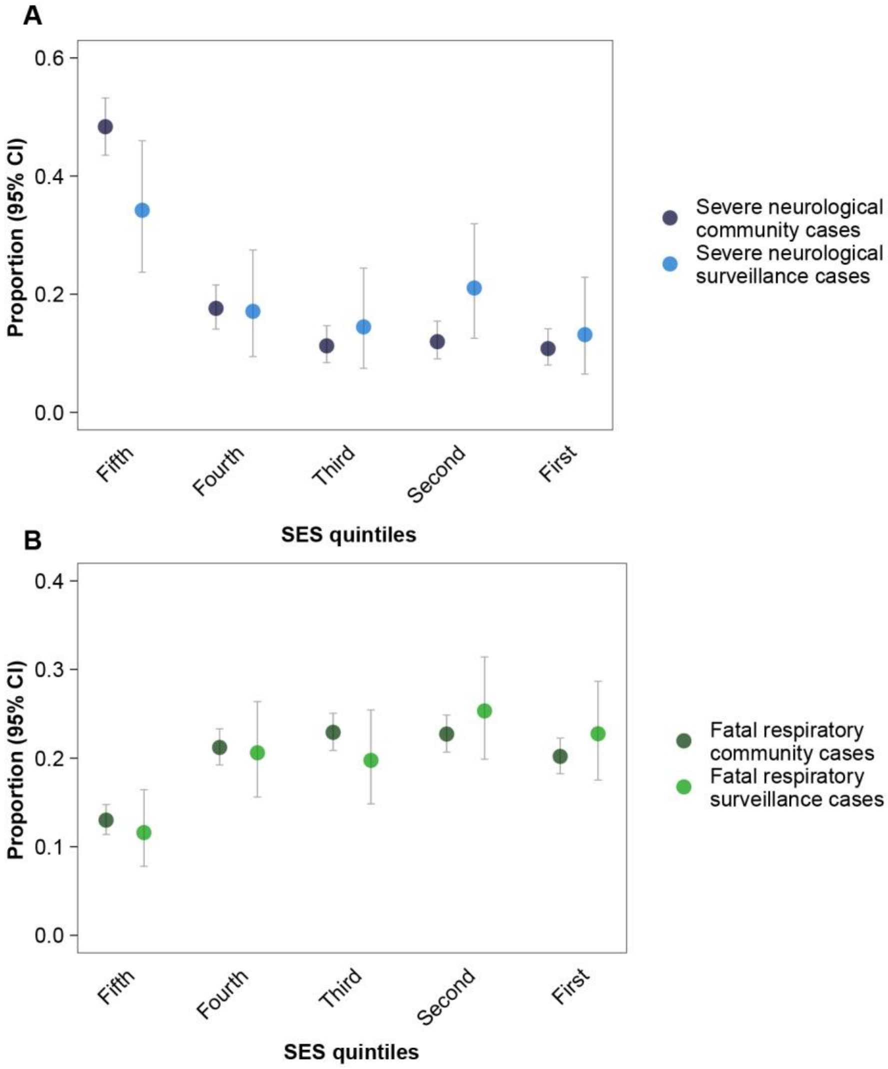

Fig E. Detection bias by socioeconomic status quintiles (the fifth quintile represents the lowest socioeconomic status group). Comparison of case statistics (proportion of characteristics) estimated based on **(A)** severe neurological and **(B)** fatal respiratory community cases to those estimated based on surveillance cases.

Representativeness of surveillance cases

	Severe neurological (n=426)				Fatal respiratory (n=1,630)			
	% among community cases	% among surveillance cases	Difference (95% CI)	p-value	% among community cases	% among surveillance cases	Difference (95% CI)	p-value
**Sex**								
Female	40	39	-1 (-11; 9)	0.861	38	34	-4 (-10; 1)	0.108
Male	60	61	1(-9; 10)	0.861	62	66	4 (-1; 10)	0.121
**Age group**								
<5	48	29	-19 (-28; -9)	<0.001	18	24	6 (1; 11)	0.020
5-14	19	22	3 (-6; 11)	0.511	1	2	1 (-1; 3)	0.313
15-59	29	43	14 (5; 25)	0.005	18	27	9 (4; 14)	<0.001
≥60	4	5	1 (-2; 7)	0.442	62	47	-15 (-22; -10)	<0.001
**Socioeconomic status**								
Lowest	57	43	-14 (-23; -3)	0.012	28	26	-2 (-8; 3)	0.385
Middle	31	39	8 (-1; 19)	0.083	35	31	-4 (-9; 2)	0.205
Highest	13	17	4 (-3; 12)	0.232	37	43	6 (0; 12)	0.046

Table B. Characteristics of all cases identified in the community and identified cases who attended a surveillance hospital. Ninety-five percent confidence intervals and p-values were obtained using bootstrap.

Outbreak detection probabilities with alternative healthcare providers

To investigate the effects of integrating additional healthcare providers in the surveillance system on the capacity to detect outbreaks, case detection probabilities by distance from the original surveillance hospitals were estimated by combining surveillance hospitals with (i) other hospitals (all other hospitals attended by cases), (ii) healthcare providers of the local formal sector, and (iii) informal healthcare providers. These case detection probabilities were used to estimate the outbreak size required to reach a 90% outbreak detection probability by distance from surveillance hospitals. Including other hospitals that were attended by cases in the surveillance system would allow detecting outbreaks (defined as at least one detected case) of four severe neurological and eight fatal respiratory cases with ≥90% probability at any distance in the range of 0-40 km from the surveillance hospital (Fig F).

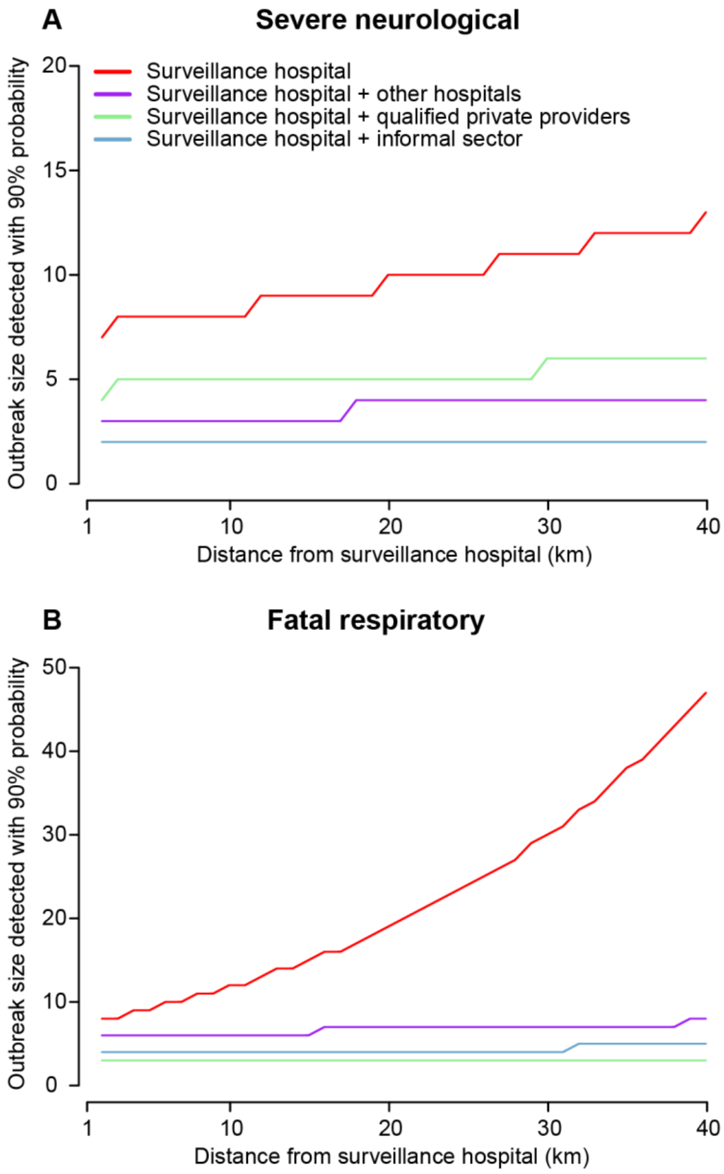

Fig F. Outbreak detection including alternative healthcare providers (outbreak threshold of at least one detected case). Sizes of **(A)** severe neurological and **(B)** fatal respiratory disease outbreaks by distance from the original surveillance hospitals, achieving a ≥90% detection probability if alternative healthcare providers were included in the surveillance system.

Representativeness of cases attending alternative healthcare providers

To assess whether including other healthcare provider classes in the surveillance system would improve the representativeness of the surveillance system, we estimated the difference between case statistics based on community cases and those based on cases attending each of the healthcare provider types (Fig G).

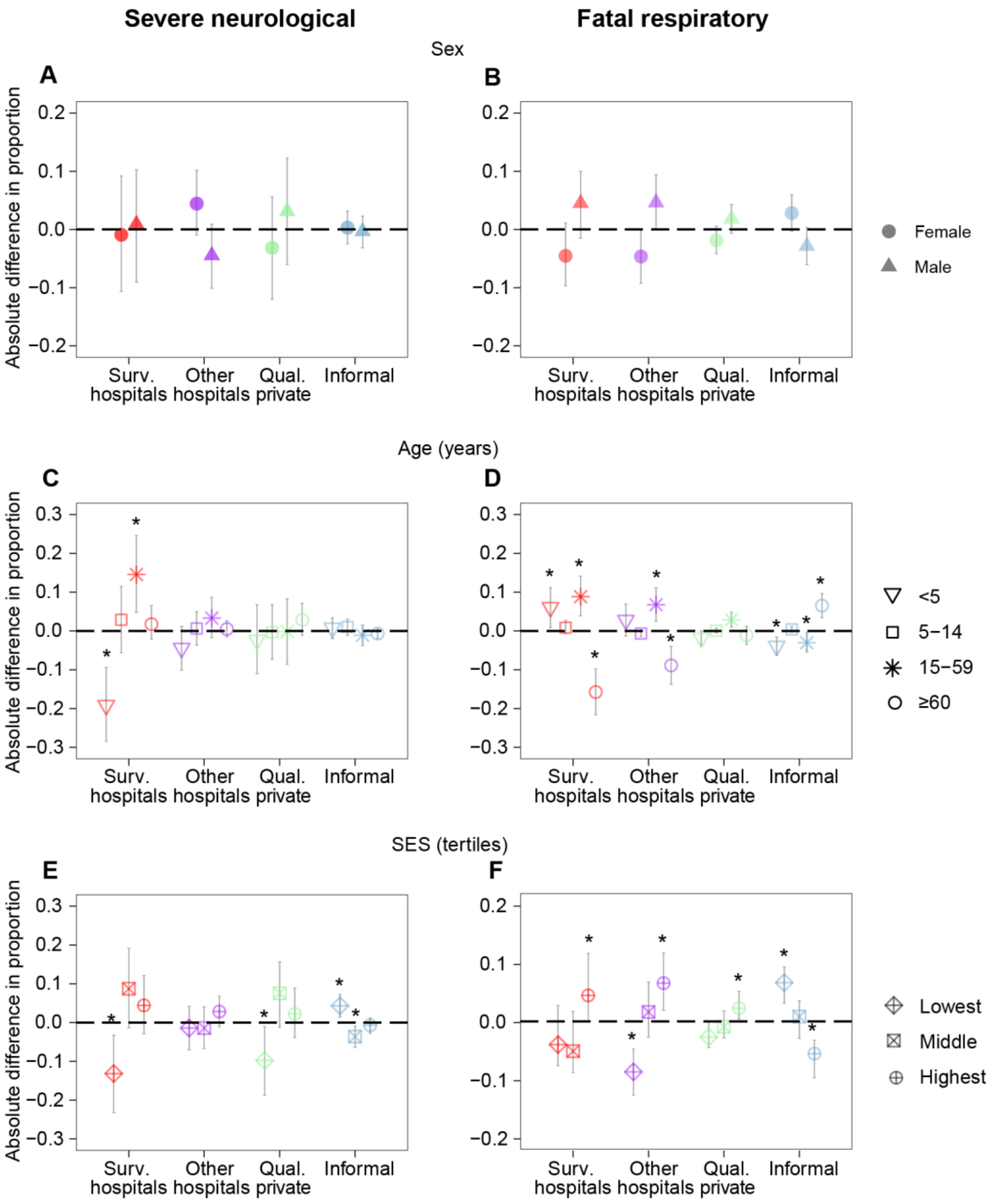

Fig G. Representativeness of cases attending other healthcare providers. Absolute difference between **(A)** severe neurological and **(B)** fatal respiratory case statistics (proportions of case characteristics) estimates based on community cases and those estimates based on cases attending each of the healthcare provider types. A negative difference indicates that proportions among cases attending a surveillance hospital are lower than among all cases in the community. Significant differences (bootstrap p≤0.05) are indicated with an asterisk (*).
